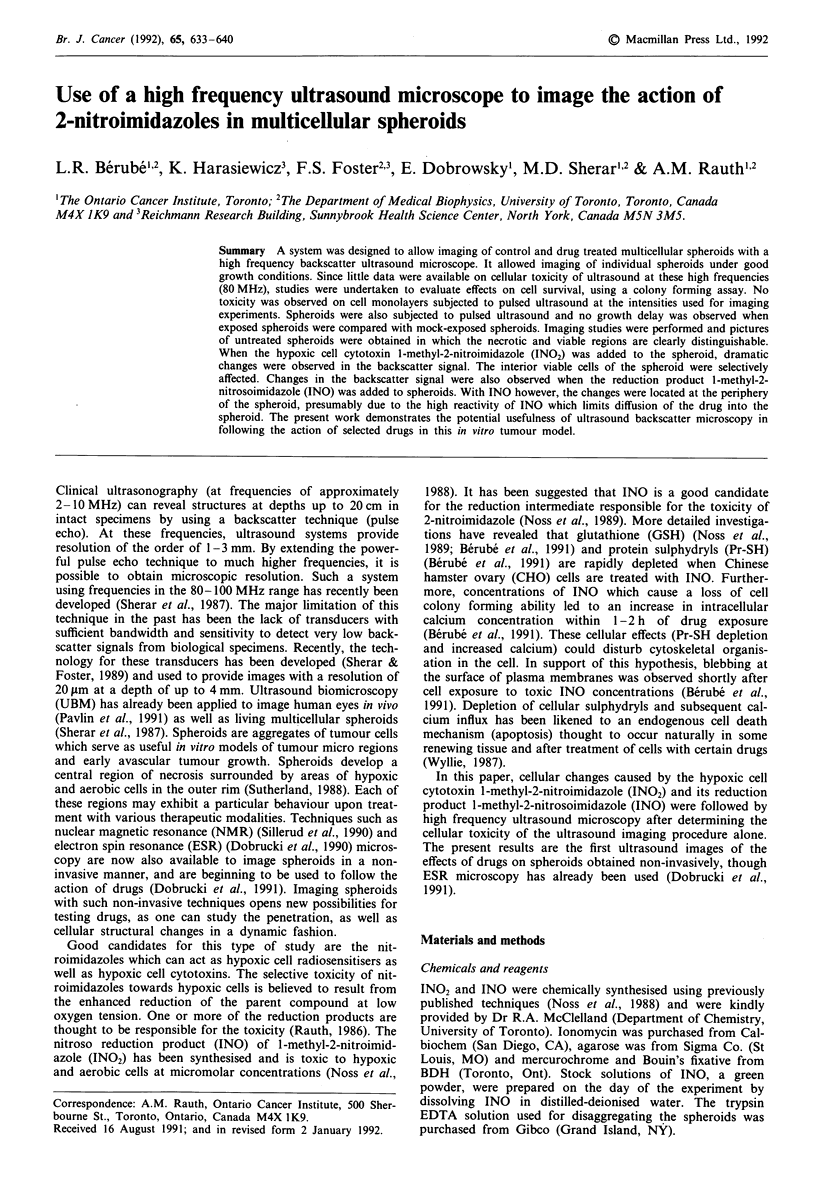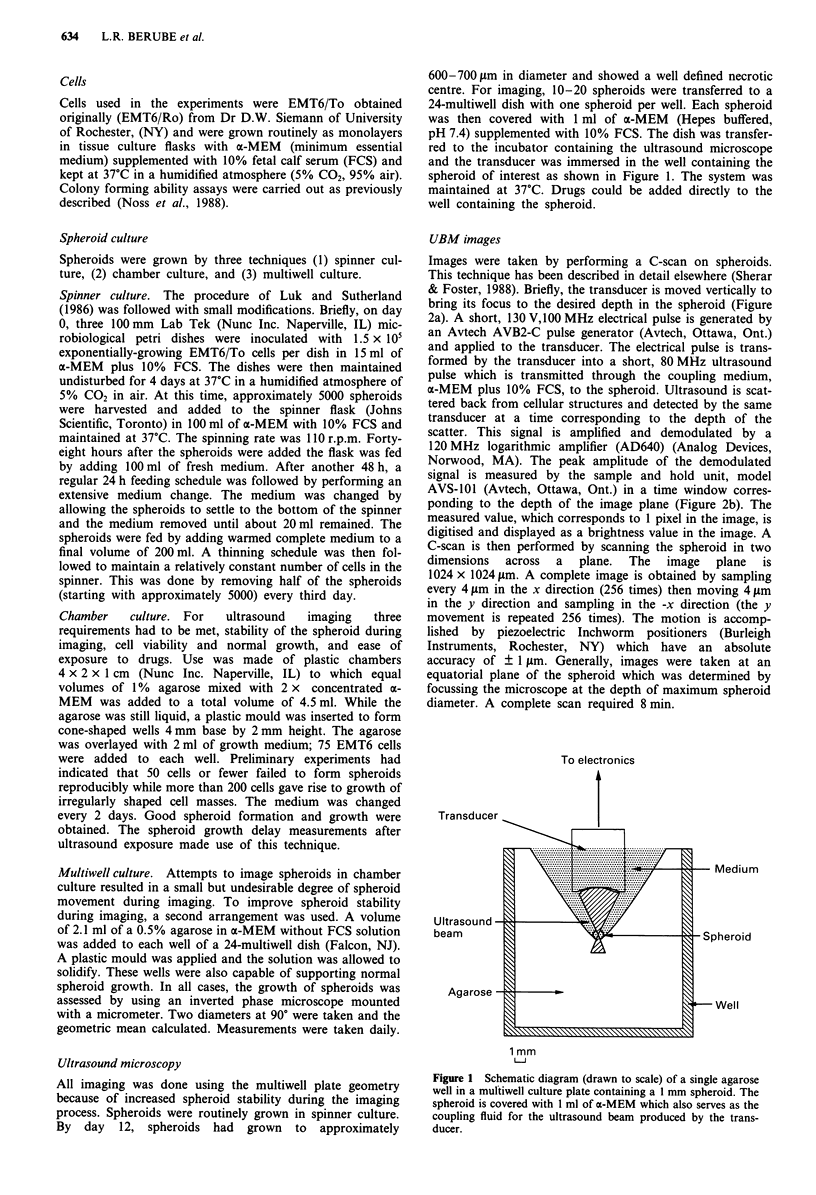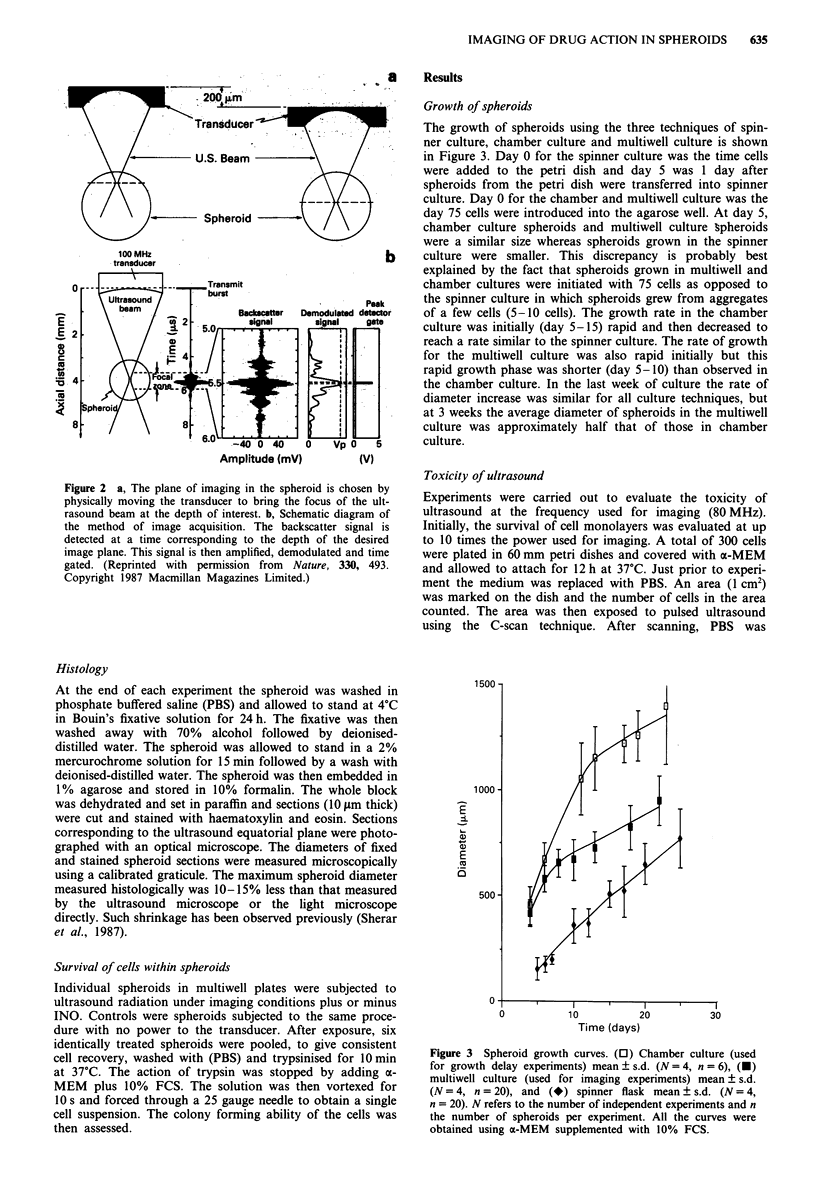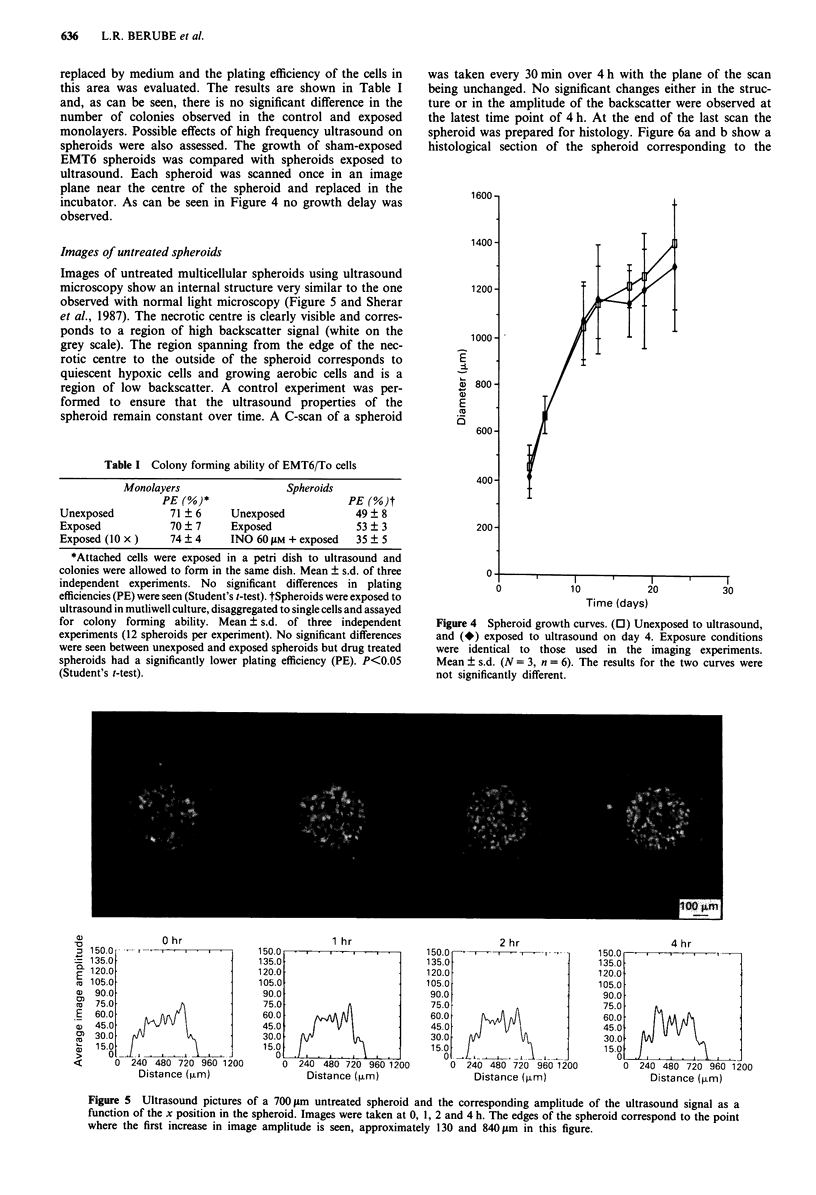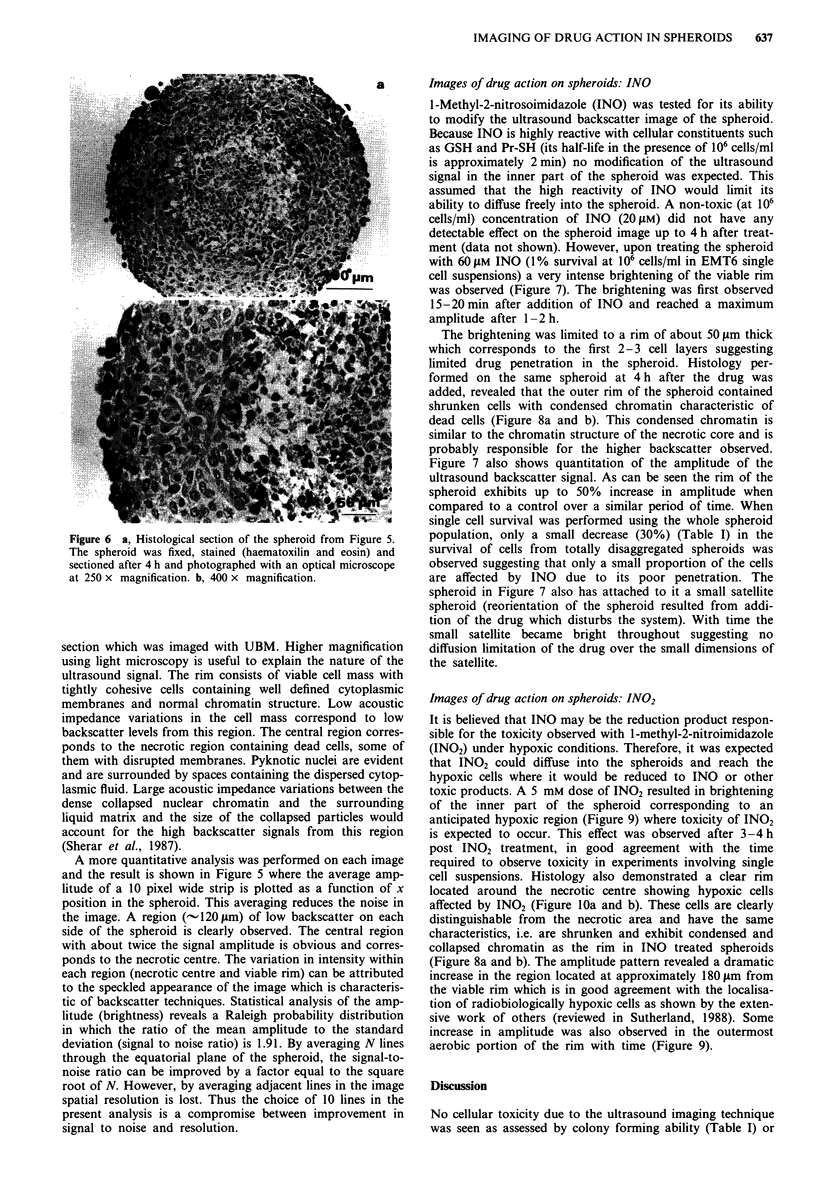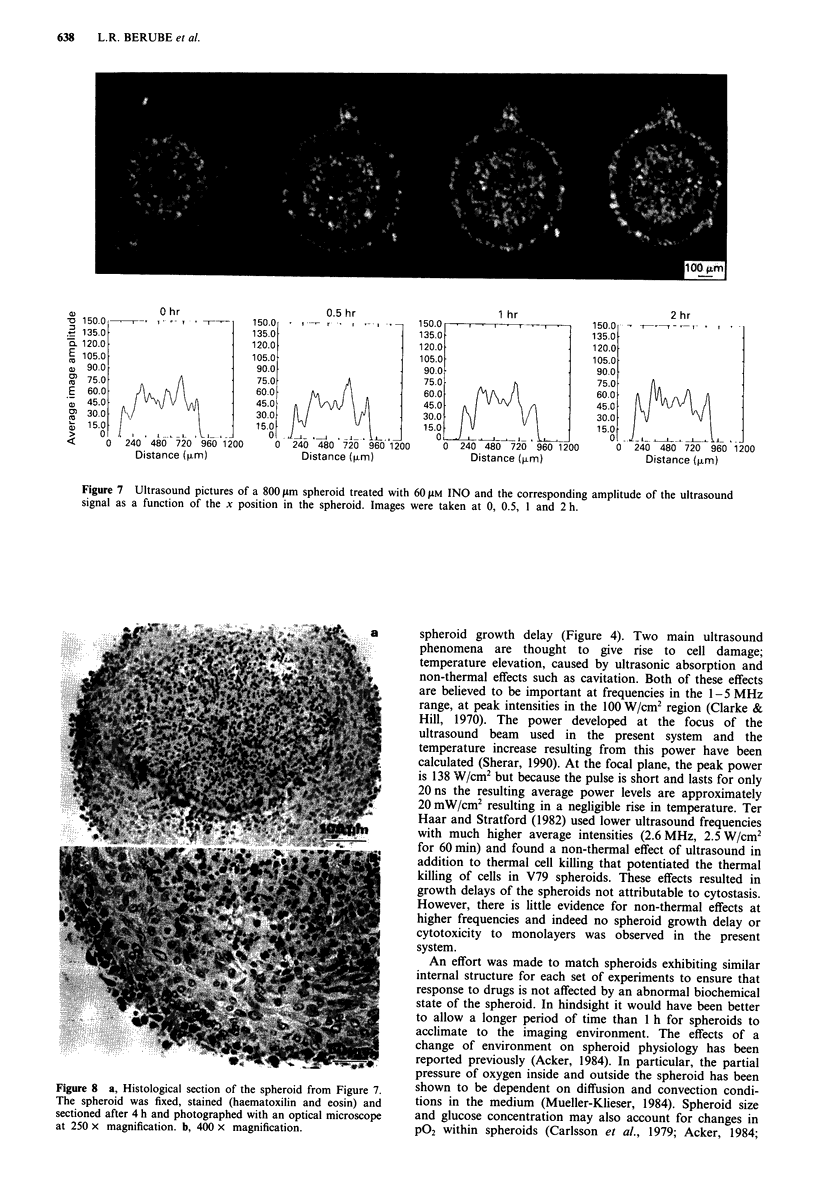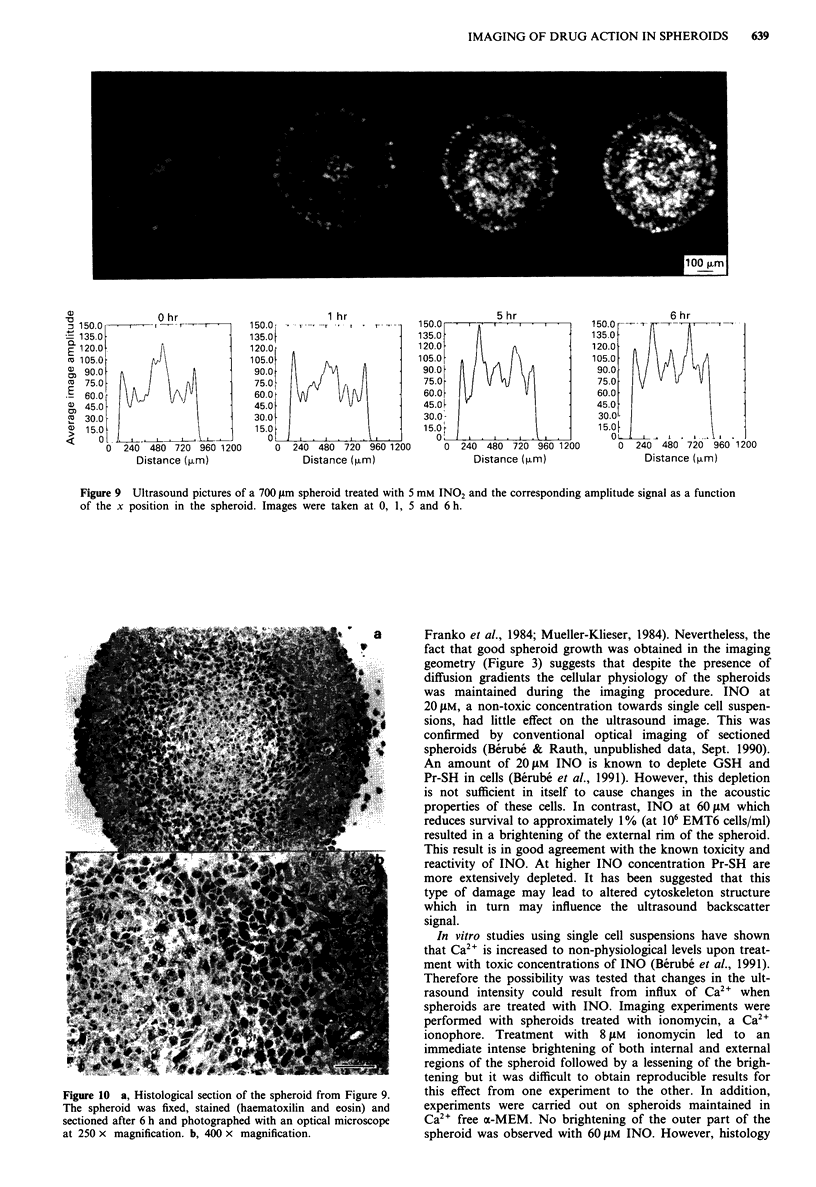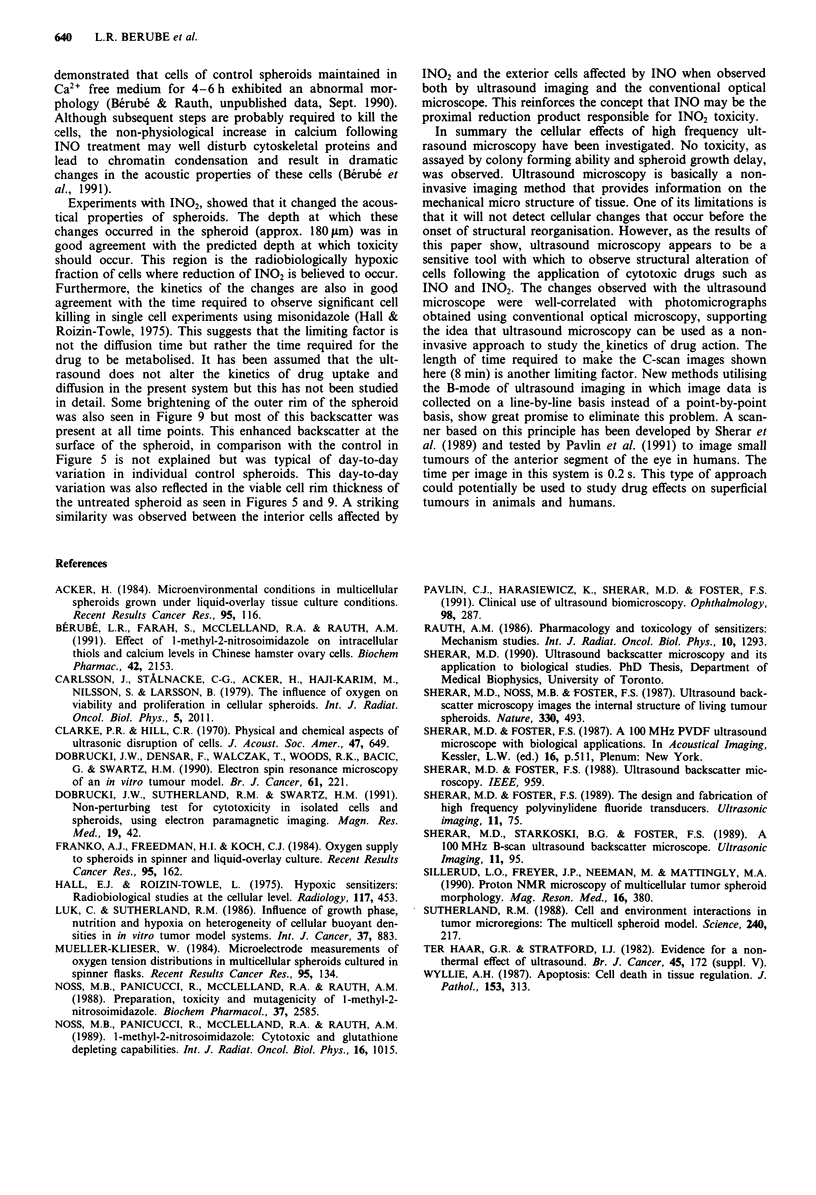# Use of a high frequency ultrasound microscope to image the action of 2-nitroimidazoles in multicellular spheroids.

**DOI:** 10.1038/bjc.1992.137

**Published:** 1992-05

**Authors:** L. R. Bérubé, K. Harasiewicz, F. S. Foster, E. Dobrowsky, M. D. Sherar, A. M. Rauth

**Affiliations:** Ontario Cancer Institute, Toronto, Canada.

## Abstract

**Images:**


Br. J. Cancer (1992), 65, 633-640 © Macmillan Press Ltd., 1992

### Use of a high frequency ultrasound microscope to image the action of 2-nitroimidazoles in multicellular spheroids

L.R. Bérubé[1,2], K. Harasiewicz[3], F.S. Foster[2,3], E. Dobrowsky[1], M.D. Sherar[1,2] & A.M. Rauth[1,2]

[1]*The Ontario Cancer Institute, Toronto;* [2]*The Department of Medical Biophysics, University of Toronto, Toronto, Canada M4X 1K9 and* [3]*Reichmann Research Building, Sunnybrook Health Science Center, North York, Canada M5N 3M5.*

**Summary** A system was designed to allow imaging of control and drug treated multicellular spheroids with a high frequency backscatter ultrasound microscope. It allowed imaging of individual spheroids under good growth conditions. Since little data were available on cellular toxicity of ultrasound at these high frequencies (80 MHz), studies were undertaken to evaluate effects on cell survival, using a colony forming assay. No toxicity was observed on cell monolayers subjected to pulsed ultrasound at the intensities used for imaging experiments. Spheroids were also subjected to pulsed ultrasound and no growth delay was observed when exposed spheroids were compared with mock-exposed spheroids. Imaging studies were performed and pictures of untreated spheroids were obtained in which the necrotic and viable regions are clearly distinguishable. When the hypoxic cell cytotoxin 1-methyl-2-nitroimidazole ($INO_2$) was added to the spheroid, dramatic changes were observed in the backscatter signal. The interior viable cells of the spheroid were selectively affected. Changes in the backscatter signal were also observed when the reduction product 1-methyl-2-nitrosoimidazole (INO) was added to spheroids. With INO however, the changes were located at the periphery of the spheroid, presumably due to the high reactivity of INO which limits diffusion of the drug into the spheroid. The present work demonstrates the potential usefulness of ultrasound backscatter microscopy in following the action of selected drugs in this *in vitro* tumour model.

Clinical ultrasonography (at frequencies of approximately 2–10 MHz) can reveal structures at depths up to 20 cm in intact specimens by using a backscatter technique (pulse echo). At these frequencies, ultrasound systems provide resolution of the order of 1–3 mm. By extending the powerful pulse echo technique to much higher frequencies, it is possible to obtain microscopic resolution. Such a system using frequencies in the 80–100 MHz range has recently been developed (Sherar *et al.*, 1987). The major limitation of this technique in the past has been the lack of transducers with sufficient bandwidth and sensitivity to detect very low backscatter signals from biological specimens. Recently, the technology for these transducers has been developed (Sherar & Foster, 1989) and used to provide images with a resolution of 20 µm at a depth of up to 4 mm. Ultrasound biomicroscopy (UBM) has already been applied to image human eyes *in vivo* (Pavlin *et al.*, 1991) as well as living multicellular spheroids (Sherar *et al.*, 1987). Spheroids are aggregates of tumour cells which serve as useful *in vitro* models of tumour micro regions and early avascular tumour growth. Spheroids develop a central region of necrosis surrounded by areas of hypoxic and aerobic cells in the outer rim (Sutherland, 1988). Each of these regions may exhibit a particular behaviour upon treatment with various therapeutic modalities. Techniques such as nuclear magnetic resonance (NMR) (Sillerud *et al.*, 1990) and electron spin resonance (ESR) (Dobrucki *et al.*, 1990) microscopy are now also available to image spheroids in a non-invasive manner, and are beginning to be used to follow the action of drugs (Dobrucki *et al.*, 1991). Imaging spheroids with such non-invasive techniques opens new possibilities for testing drugs, as one can study the penetration, as well as cellular structural changes in a dynamic fashion.

Good candidates for this type of study are the nitroimidazoles which can act as hypoxic cell radiosensitisers as well as hypoxic cell cytotoxins. The selective toxicity of nitroimidazoles towards hypoxic cells is believed to result from the enhanced reduction of the parent compound at low oxygen tension. One or more of the reduction products are thought to be responsible for the toxicity (Rauth, 1986). The nitroso reduction product (INO) of 1-methyl-2-nitroimidazole ($INO_2$) has been synthesised and is toxic to hypoxic and aerobic cells at micromolar concentrations (Noss *et al.*, 1988). It has been suggested that INO is a good candidate for the reduction intermediate responsible for the toxicity of 2-nitroimidazole (Noss *et al.*, 1989). More detailed investigations have revealed that glutathione (GSH) (Noss *et al.*, 1989; Bérubé *et al.*, 1991) and protein sulphydryls (Pr-SH) (Bérubé *et al.*, 1991) are rapidly depleted when Chinese hamster ovary (CHO) cells are treated with INO. Furthermore, concentrations of INO which cause a loss of cell colony forming ability led to an increase in intracellular calcium concentration within 1–2 h of drug exposure (Bérubé *et al.*, 1991). These cellular effects (Pr-SH depletion and increased calcium) could disturb cytoskeletal organisation in the cell. In support of this hypothesis, blebbing at the surface of plasma membranes was observed shortly after cell exposure to toxic INO concentrations (Bérubé *et al.*, 1991). Depletion of cellular sulphydryls and subsequent calcium influx has been likened to an endogenous cell death mechanism (apoptosis) thought to occur naturally in some renewing tissue and after treatment of cells with certain drugs (Wyllie, 1987).

In this paper, cellular changes caused by the hypoxic cell cytotoxin 1-methyl-2-nitroimidazole ($INO_2$) and its reduction product 1-methyl-2-nitrosoimidazole (INO) were followed by high frequency ultrasound microscopy after determining the cellular toxicity of the ultrasound imaging procedure alone. The present results are the first ultrasound images of the effects of drugs on spheroids obtained non-invasively, though ESR microscopy has already been used (Dobrucki *et al.*, 1991).

Correspondence: A.M. Rauth, Ontario Cancer Institute, 500 Sherbourne St., Toronto, Ontario, Canada M4X 1K9.
Received 16 August 1991; and in revised form 2 January 1992.

### Materials and methods

### Chemicals and reagents

$INO_2$ and INO were chemically synthesised using previously published techniques (Noss *et al.*, 1988) and were kindly provided by Dr R.A. McClelland (Department of Chemistry, University of Toronto). Ionomycin was purchased from Calbiochem (San Diego, CA), agarose was from Sigma Co. (St Louis, MO) and mercurochrome and Bouin's fixative from BDH (Toronto, Ont). Stock solutions of INO, a green powder, were prepared on the day of the experiment by dissolving INO in distilled-deionised water. The trypsin EDTA solution used for disaggregating the spheroids was purchased from Gibco (Grand Island, NY).

*Cells*

Cells used in the experiments were EMT6/To obtained originally (EMT6/Ro) from Dr D.W. Siemann of University of Rochester, (NY) and were grown routinely as monolayers in tissue culture flasks with α-MEM (minimum essential medium) supplemented with 10% fetal calf serum (FCS) and kept at 37°C in a humidified atmosphere (5% $CO_2$, 95% air). Colony forming ability assays were carried out as previously described (Noss *et al.*, 1988).

*Spheroid culture*

Spheroids were grown by three techniques (1) spinner culture, (2) chamber culture, and (3) multiwell culture.

*Spinner culture.* The procedure of Luk and Sutherland (1986) was followed with small modifications. Briefly, on day 0, three 100 mm Lab Tek (Nunc Inc. Naperville, IL) microbiological petri dishes were inoculated with $1.5 \times 10^5$ exponentially-growing EMT6/To cells per dish in 15 ml of α-MEM plus 10% FCS. The dishes were then maintained undisturbed for 4 days at 37°C in a humidified atmosphere of 5% $CO_2$ in air. At this time, approximately 5000 spheroids were harvested and added to the spinner flask (Johns Scientific, Toronto) in 100 ml of α-MEM with 10% FCS and maintained at 37°C. The spinning rate was 110 r.p.m. Forty-eight hours after the spheroids were added the flask was fed by adding 100 ml of fresh medium. After another 48 h, a regular 24 h feeding schedule was followed by performing an extensive medium change. The medium was changed by allowing the spheroids to settle to the bottom of the spinner and the medium removed until about 20 ml remained. The spheroids were fed by adding warmed complete medium to a final volume of 200 ml. A thinning schedule was then followed to maintain a relatively constant number of cells in the spinner. This was done by removing half of the spheroids (starting with approximately 5000) every third day.

*Chamber culture.* For ultrasound imaging three requirements had to be met, stability of the spheroid during imaging, cell viability and normal growth, and ease of exposure to drugs. Use was made of plastic chambers 4 × 2 × 1 cm (Nunc Inc. Naperville, IL) to which equal volumes of 1% agarose mixed with 2 × concentrated α-MEM was added to a total volume of 4.5 ml. While the agarose was still liquid, a plastic mould was inserted to form cone-shaped wells 4 mm base by 2 mm height. The agarose was overlayed with 2 ml of growth medium; 75 EMT6 cells were added to each well. Preliminary experiments had indicated that 50 cells or fewer failed to form spheroids reproducibly while more than 200 cells gave rise to growth of irregularly shaped cell masses. The medium was changed every 2 days. Good spheroid formation and growth were obtained. The spheroid growth delay measurements after ultrasound exposure made use of this technique.

*Multiwell culture.* Attempts to image spheroids in chamber culture resulted in a small but undesirable degree of spheroid movement during imaging. To improve spheroid stability during imaging, a second arrangement was used. A volume of 2.1 ml of a 0.5% agarose in α-MEM without FCS solution was added to each well of a 24-multiwell dish (Falcon, NJ). A plastic mould was applied and the solution was allowed to solidify. These wells were also capable of supporting normal spheroid growth. In all cases, the growth of spheroids was assessed by using an inverted phase microscope mounted with a micrometer. Two diameters at 90° were taken and the geometric mean calculated. Measurements were taken daily.

*Ultrasound microscopy*

All imaging was done using the multiwell plate geometry because of increased spheroid stability during the imaging process. Spheroids were routinely grown in spinner culture. By day 12, spheroids had grown to approximately 600–700 μm in diameter and showed a well defined necrotic centre. For imaging, 10–20 spheroids were transferred to a 24-multiwell dish with one spheroid per well. Each spheroid was then covered with 1 ml of α-MEM (Hepes buffered, pH 7.4) supplemented with 10% FCS. The dish was transferred to the incubator containing the ultrasound microscope and the transducer was immersed in the well containing the spheroid of interest as shown in Figure 1. The system was maintained at 37°C. Drugs could be added directly to the well containing the spheroid.

*UBM images*

Images were taken by performing a C-scan on spheroids. This technique has been described in detail elsewhere (Sherar & Foster, 1988). Briefly, the transducer is moved vertically to bring its focus to the desired depth in the spheroid (Figure 2a). A short, 130 V,100 MHz electrical pulse is generated by an Avtech AVB2-C pulse generator (Avtech, Ottawa, Ont.) and applied to the transducer. The electrical pulse is transformed by the transducer into a short, 80 MHz ultrasound pulse which is transmitted through the coupling medium, α-MEM plus 10% FCS, to the spheroid. Ultrasound is scattered back from cellular structures and detected by the same transducer at a time corresponding to the depth of the scatter. This signal is amplified and demodulated by a 120 MHz logarithmic amplifier (AD640) (Analog Devices, Norwood, MA). The peak amplitude of the demodulated signal is measured by the sample and hold unit, model AVS-101 (Avtech, Ottawa, Ont.) in a time window corresponding to the depth of the image plane (Figure 2b). The measured value, which corresponds to 1 pixel in the image, is digitised and displayed as a brightness value in the image. A C-scan is then performed by scanning the spheroid in two dimensions across a plane. The image plane is 1024 × 1024 μm. A complete image is obtained by sampling every 4 μm in the $x$ direction (256 times) then moving 4 μm in the $y$ direction and sampling in the $-x$ direction (the $y$ movement is repeated 256 times). The motion is accomplished by piezoelectric Inchworm positioners (Burleigh Instruments, Rochester, NY) which have an absolute accuracy of ± 1 μm. Generally, images were taken at an equatorial plane of the spheroid which was determined by focussing the microscope at the depth of maximum spheroid diameter. A complete scan required 8 min.

**Figure 1** Schematic diagram (drawn to scale) of a single agarose well in a multiwell culture plate containing a 1 mm spheroid. The spheroid is covered with 1 ml of α-MEM which also serves as the coupling fluid for the ultrasound beam produced by the transducer.

**Figure 2** **a**, The plane of imaging in the spheroid is chosen by physically moving the transducer to bring the focus of the ultrasound beam at the depth of interest. **b**, Schematic diagram of the method of image acquisition. The backscatter signal is detected at a time corresponding to the depth of the desired image plane. This signal is then amplified, demodulated and time gated. (Reprinted with permission from *Nature*, **330**, 493. Copyright 1987 Macmillan Magazines Limited.)

*Histology*

At the end of each experiment the spheroid was washed in phosphate buffered saline (PBS) and allowed to stand at 4°C in Bouin's fixative solution for 24 h. The fixative was then washed away with 70% alcohol followed by deionised-distilled water. The spheroid was allowed to stand in a 2% mercurochrome solution for 15 min followed by a wash with deionised-distilled water. The spheroid was then embedded in 1% agarose and stored in 10% formalin. The whole block was dehydrated and set in paraffin and sections (10 µm thick) were cut and stained with haematoxylin and eosin. Sections corresponding to the ultrasound equatorial plane were photographed with an optical microscope. The diameters of fixed and stained spheroid sections were measured microscopically using a calibrated graticule. The maximum spheroid diameter measured histologically was 10–15% less than that measured by the ultrasound microscope or the light microscope directly. Such shrinkage has been observed previously (Sherar *et al.*, 1987).

*Survival of cells within spheroids*

Individual spheroids in multiwell plates were subjected to ultrasound radiation under imaging conditions plus or minus INO. Controls were spheroids subjected to the same procedure with no power to the transducer. After exposure, six identically treated spheroids were pooled, to give consistent cell recovery, washed with (PBS) and trypsinised for 10 min at 37°C. The action of trypsin was stopped by adding α-MEM plus 10% FCS. The solution was then vortexed for 10 s and forced through a 25 gauge needle to obtain a single cell suspension. The colony forming ability of the cells was then assessed.

### Results

*Growth of spheroids*

The growth of spheroids using the three techniques of spinner culture, chamber culture and multiwell culture is shown in Figure 3. Day 0 for the spinner culture was the time cells were added to the petri dish and day 5 was 1 day after spheroids from the petri dish were transferred into spinner culture. Day 0 for the chamber and multiwell culture was the day 75 cells were introduced into the agarose well. At day 5, chamber culture spheroids and multiwell culture spheroids were a similar size whereas spheroids grown in the spinner culture were smaller. This discrepancy is probably best explained by the fact that spheroids grown in multiwell and chamber cultures were initiated with 75 cells as opposed to the spinner culture in which spheroids grew from aggregates of a few cells (5–10 cells). The growth rate in the chamber culture was initially (day 5–15) rapid and then decreased to reach a rate similar to the spinner culture. The rate of growth for the multiwell culture was also rapid initially but this rapid growth phase was shorter (day 5–10) than observed in the chamber culture. In the last week of culture the rate of diameter increase was similar for all culture techniques, but at 3 weeks the average diameter of spheroids in the multiwell culture was approximately half that of those in chamber culture.

*Toxicity of ultrasound*

Experiments were carried out to evaluate the toxicity of ultrasound at the frequency used for imaging (80 MHz). Initially, the survival of cell monolayers was evaluated at up to 10 times the power used for imaging. A total of 300 cells were plated in 60 mm petri dishes and covered with α-MEM and allowed to attach for 12 h at 37°C. Just prior to experiment the medium was replaced with PBS. An area (1 $cm^2$) was marked on the dish and the number of cells in the area counted. The area was then exposed to pulsed ultrasound using the C-scan technique. After scanning, PBS was

**Figure 3** Spheroid growth curves. (□) Chamber culture (used for growth delay experiments) mean ± s.d. ($N = 4$, $n = 6$), (■) multiwell culture (used for imaging experiments) mean ± s.d. ($N = 4$, $n = 20$), and (◆) spinner flask mean ± s.d. ($N = 4$, $n = 20$). $N$ refers to the number of independent experiments and $n$ the number of spheroids per experiment. All the curves were obtained using α-MEM supplemented with 10% FCS.

replaced by medium and the plating efficiency of the cells in this area was evaluated. The results are shown in Table I and, as can be seen, there is no significant difference in the number of colonies observed in the control and exposed monolayers. Possible effects of high frequency ultrasound on spheroids were also assessed. The growth of sham-exposed EMT6 spheroids was compared with spheroids exposed to ultrasound. Each spheroid was scanned once in an image plane near the centre of the spheroid and replaced in the incubator. As can be seen in Figure 4 no growth delay was observed.

### *Images of untreated spheroids*

Images of untreated multicellular spheroids using ultrasound microscopy show an internal structure very similar to the one observed with normal light microscopy (Figure 5 and Sherar *et al.*, 1987). The necrotic centre is clearly visible and corresponds to a region of high backscatter signal (white on the grey scale). The region spanning from the edge of the necrotic centre to the outside of the spheroid corresponds to quiescent hypoxic cells and growing aerobic cells and is a region of low backscatter. A control experiment was performed to ensure that the ultrasound properties of the spheroid remain constant over time. A C-scan of a spheroid was taken every 30 min over 4 h with the plane of the scan being unchanged. No significant changes either in the structure or in the amplitude of the backscatter were observed at the latest time point of 4 h. At the end of the last scan the spheroid was prepared for histology. Figure 6a and b show a histological section of the spheroid corresponding to the

**Table I** Colony forming ability of EMT6/To cells

| *Monolayers* | | *Spheroids* | |
|---|---|---|---|
| | *PE (%)** | | *PE (%)*† |
| Unexposed | 71 ± 6 | Unexposed | 49 ± 8 |
| Exposed | 70 ± 7 | Exposed | 53 ± 3 |
| Exposed (10 ×) | 74 ± 4 | INO 60 μM + exposed | 35 ± 5 |

*Attached cells were exposed in a petri dish to ultrasound and colonies were allowed to form in the same dish. Mean ± s.d. of three independent experiments. No significant differences in plating efficiencies (PE) were seen (Student's *t*-test). †Spheroids were exposed to ultrasound in mutliwell culture, disaggregated to single cells and assayed for colony forming ability. Mean ± s.d. of three independent experiments (12 spheroids per experiment). No significant differences were seen between unexposed and exposed spheroids but drug treated spheroids had a significantly lower plating efficiency (PE). $P<0.05$ (Student's *t*-test).

**Figure 4** Spheroid growth curves. (□) Unexposed to ultrasound, and (◆) exposed to ultrasound on day 4. Exposure conditions were identical to those used in the imaging experiments. Mean ± s.d. ($N = 3$, $n = 6$). The results for the two curves were not significantly different.

**Figure 5** Ultrasound pictures of a 700 μm untreated spheroid and the corresponding amplitude of the ultrasound signal as a function of the *x* position in the spheroid. Images were taken at 0, 1, 2 and 4 h. The edges of the spheroid correspond to the point where the first increase in image amplitude is seen, approximately 130 and 840 μm in this figure.

**Figure 6** **a**, Histological section of the spheroid from Figure 5. The spheroid was fixed, stained (haematoxilin and eosin) and sectioned after 4 h and photographed with an optical microscope at 250 × magnification. **b**, 400 × magnification.

section which was imaged with UBM. Higher magnification using light microscopy is useful to explain the nature of the ultrasound signal. The rim consists of viable cell mass with tightly cohesive cells containing well defined cytoplasmic membranes and normal chromatin structure. Low acoustic impedance variations in the cell mass correspond to low backscatter levels from this region. The central region corresponds to the necrotic region containing dead cells, some of them with disrupted membranes. Pyknotic nuclei are evident and are surrounded by spaces containing the dispersed cytoplasmic fluid. Large acoustic impedance variations between the dense collapsed nuclear chromatin and the surrounding liquid matrix and the size of the collapsed particles would account for the high backscatter signals from this region (Sherar *et al.*, 1987).

A more quantitative analysis was performed on each image and the result is shown in Figure 5 where the average amplitude of a 10 pixel wide strip is plotted as a function of $x$ position in the spheroid. This averaging reduces the noise in the image. A region (~120 µm) of low backscatter on each side of the spheroid is clearly observed. The central region with about twice the signal amplitude is obvious and corresponds to the necrotic centre. The variation in intensity within each region (necrotic centre and viable rim) can be attributed to the speckled appearance of the image which is characteristic of backscatter techniques. Statistical analysis of the amplitude (brightness) reveals a Raleigh probability distribution in which the ratio of the mean amplitude to the standard deviation (signal to noise ratio) is 1.91. By averaging $N$ lines through the equatorial plane of the spheroid, the signal-to-noise ratio can be improved by a factor equal to the square root of $N$. However, by averaging adjacent lines in the image spatial resolution is lost. Thus the choice of 10 lines in the present analysis is a compromise between improvement in signal to noise and resolution.

*Images of drug action on spheroids: INO*

1-Methyl-2-nitrosoimidazole (INO) was tested for its ability to modify the ultrasound backscatter image of the spheroid. Because INO is highly reactive with cellular constituents such as GSH and Pr-SH (its half-life in the presence of $10^6$ cells/ml is approximately 2 min) no modification of the ultrasound signal in the inner part of the spheroid was expected. This assumed that the high reactivity of INO would limit its ability to diffuse freely into the spheroid. A non-toxic (at $10^6$ cells/ml) concentration of INO (20 µM) did not have any detectable effect on the spheroid image up to 4 h after treatment (data not shown). However, upon treating the spheroid with 60 µM INO (1% survival at $10^6$ cells/ml in EMT6 single cell suspensions) a very intense brightening of the viable rim was observed (Figure 7). The brightening was first observed 15–20 min after addition of INO and reached a maximum amplitude after 1–2 h.

The brightening was limited to a rim of about 50 µm thick which corresponds to the first 2–3 cell layers suggesting limited drug penetration in the spheroid. Histology performed on the same spheroid at 4 h after the drug was added, revealed that the outer rim of the spheroid contained shrunken cells with condensed chromatin characteristic of dead cells (Figure 8a and b). This condensed chromatin is similar to the chromatin structure of the necrotic core and is probably responsible for the higher backscatter observed. Figure 7 also shows quantitation of the amplitude of the ultrasound backscatter signal. As can be seen the rim of the spheroid exhibits up to 50% increase in amplitude when compared to a control over a similar period of time. When single cell survival was performed using the whole spheroid population, only a small decrease (30%) (Table I) in the survival of cells from totally disaggregated spheroids was observed suggesting that only a small proportion of the cells are affected by INO due to its poor penetration. The spheroid in Figure 7 also has attached to it a small satellite spheroid (reorientation of the spheroid resulted from addition of the drug which disturbs the system). With time the small satellite became bright throughout suggesting no diffusion limitation of the drug over the small dimensions of the satellite.

*Images of drug action on spheroids: $INO_2$*

It is believed that INO may be the reduction product responsible for the toxicity observed with 1-methyl-2-nitroimidazole ($INO_2$) under hypoxic conditions. Therefore, it was expected that $INO_2$ could diffuse into the spheroids and reach the hypoxic cells where it would be reduced to INO or other toxic products. A 5 mM dose of $INO_2$ resulted in brightening of the inner part of the spheroid corresponding to an anticipated hypoxic region (Figure 9) where toxicity of $INO_2$ is expected to occur. This effect was observed after 3–4 h post $INO_2$ treatment, in good agreement with the time required to observe toxicity in experiments involving single cell suspensions. Histology also demonstrated a clear rim located around the necrotic centre showing hypoxic cells affected by $INO_2$ (Figure 10a and b). These cells are clearly distinguishable from the necrotic area and have the same characteristics, i.e. are shrunken and exhibit condensed and collapsed chromatin as the rim in INO treated spheroids (Figure 8a and b). The amplitude pattern revealed a dramatic increase in the region located at approximately 180 µm from the viable rim which is in good agreement with the localisation of radiobiologically hypoxic cells as shown by the extensive work of others (reviewed in Sutherland, 1988). Some increase in amplitude was also observed in the outermost aerobic portion of the rim with time (Figure 9).

### Discussion

No cellular toxicity due to the ultrasound imaging technique was seen as assessed by colony forming ability (Table I) or

**Figure 7** Ultrasound pictures of a 800 μm spheroid treated with 60 μM INO and the corresponding amplitude of the ultrasound signal as a function of the $x$ position in the spheroid. Images were taken at 0, 0.5, 1 and 2 h.

**Figure 8** **a**, Histological section of the spheroid from Figure 7. The spheroid was fixed, stained (haematoxilin and eosin) and sectioned after 4 h and photographed with an optical microscope at 250 × magnification. **b**, 400 × magnification.

spheroid growth delay (Figure 4). Two main ultrasound phenomena are thought to give rise to cell damage; temperature elevation, caused by ultrasonic absorption and non-thermal effects such as cavitation. Both of these effects are believed to be important at frequencies in the 1–5 MHz range, at peak intensities in the 100 W/cm$^2$ region (Clarke & Hill, 1970). The power developed at the focus of the ultrasound beam used in the present system and the temperature increase resulting from this power have been calculated (Sherar, 1990). At the focal plane, the peak power is 138 W/cm$^2$ but because the pulse is short and lasts for only 20 ns the resulting average power levels are approximately 20 mW/cm$^2$ resulting in a negligible rise in temperature. Ter Haar and Stratford (1982) used lower ultrasound frequencies with much higher average intensities (2.6 MHz, 2.5 W/cm$^2$ for 60 min) and found a non-thermal effect of ultrasound in addition to thermal cell killing that potentiated the thermal killing of cells in V79 spheroids. These effects resulted in growth delays of the spheroids not attributable to cytostasis. However, there is little evidence for non-thermal effects at higher frequencies and indeed no spheroid growth delay or cytotoxicity to monolayers was observed in the present system.

An effort was made to match spheroids exhibiting similar internal structure for each set of experiments to ensure that response to drugs is not affected by an abnormal biochemical state of the spheroid. In hindsight it would have been better to allow a longer period of time than 1 h for spheroids to acclimate to the imaging environment. The effects of a change of environment on spheroid physiology has been reported previously (Acker, 1984). In particular, the partial pressure of oxygen inside and outside the spheroid has been shown to be dependent on diffusion and convection conditions in the medium (Mueller-Klieser, 1984). Spheroid size and glucose concentration may also account for changes in $pO_2$ within spheroids (Carlsson *et al.*, 1979; Acker, 1984;

**Figure 9** Ultrasound pictures of a 700 μm spheroid treated with 5 mM $INO_2$ and the corresponding amplitude signal as a function of the $x$ position in the spheroid. Images were taken at 0, 1, 5 and 6 h.

**Figure 10** **a**, Histological section of the spheroid from Figure 9. The spheroid was fixed, stained (haematoxilin and eosin) and sectioned after 6 h and photographed with an optical microscope at 250 × magnification. **b**, 400 × magnification.

Franko *et al.*, 1984; Mueller-Klieser, 1984). Nevertheless, the fact that good spheroid growth was obtained in the imaging geometry (Figure 3) suggests that despite the presence of diffusion gradients the cellular physiology of the spheroids was maintained during the imaging procedure. INO at 20 μM, a non-toxic concentration towards single cell suspensions, had little effect on the ultrasound image. This was confirmed by conventional optical imaging of sectioned spheroids (Bérubé & Rauth, unpublished data, Sept. 1990). An amount of 20 μM INO is known to deplete GSH and Pr-SH in cells (Bérubé *et al.*, 1991). However, this depletion is not sufficient in itself to cause changes in the acoustic properties of these cells. In contrast, INO at 60 μM which reduces survival to approximately 1% (at $10^6$ EMT6 cells/ml) resulted in a brightening of the external rim of the spheroid. This result is in good agreement with the known toxicity and reactivity of INO. At higher INO concentration Pr-SH are more extensively depleted. It has been suggested that this type of damage may lead to altered cytoskeleton structure which in turn may influence the ultrasound backscatter signal.

*In vitro* studies using single cell suspensions have shown that $Ca^{2+}$ is increased to non-physiological levels upon treatment with toxic concentrations of INO (Bérubé *et al.*, 1991). Therefore the possibility was tested that changes in the ultrasound intensity could result from influx of $Ca^{2+}$ when spheroids are treated with INO. Imaging experiments were performed with spheroids treated with ionomycin, a $Ca^{2+}$ ionophore. Treatment with 8 μM ionomycin led to an immediate intense brightening of both internal and external regions of the spheroid followed by a lessening of the brightening but it was difficult to obtain reproducible results for this effect from one experiment to the other. In addition, experiments were carried out on spheroids maintained in $Ca^{2+}$ free α-MEM. No brightening of the outer part of the spheroid was observed with 60 μM INO. However, histology demonstrated that cells of control spheroids maintained in $Ca^{2+}$ free medium for 4–6 h exhibited an abnormal morphology (Bérubé & Rauth, unpublished data, Sept. 1990). Although subsequent steps are probably required to kill the cells, the non-physiological increase in calcium following INO treatment may well disturb cytoskeletal proteins and lead to chromatin condensation and result in dramatic changes in the acoustic properties of these cells (Bérubé *et al.*, 1991).

Experiments with $INO_2$, showed that it changed the acoustical properties of spheroids. The depth at which these changes occurred in the spheroid (approx. 180 μm) was in good agreement with the predicted depth at which toxicity should occur. This region is the radiobiologically hypoxic fraction of cells where reduction of $INO_2$ is believed to occur. Furthermore, the kinetics of the changes are also in good agreement with the time required to observe significant cell killing in single cell experiments using misonidazole (Hall & Roizin-Towle, 1975). This suggests that the limiting factor is not the diffusion time but rather the time required for the drug to be metabolised. It has been assumed that the ultrasound does not alter the kinetics of drug uptake and diffusion in the present system but this has not been studied in detail. Some brightening of the outer rim of the spheroid was also seen in Figure 9 but most of this backscatter was present at all time points. This enhanced backscatter at the surface of the spheroid, in comparison with the control in Figure 5 is not explained but was typical of day-to-day variation in individual control spheroids. This day-to-day variation was also reflected in the viable cell rim thickness of the untreated spheroid as seen in Figures 5 and 9. A striking similarity was observed between the interior cells affected by $INO_2$ and the exterior cells affected by INO when observed both by ultrasound imaging and the conventional optical microscope. This reinforces the concept that INO may be the proximal reduction product responsible for $INO_2$ toxicity.

In summary the cellular effects of high frequency ultrasound microscopy have been investigated. No toxicity, as assayed by colony forming ability and spheroid growth delay, was observed. Ultrasound microscopy is basically a non-invasive imaging method that provides information on the mechanical micro structure of tissue. One of its limitations is that it will not detect cellular changes that occur before the onset of structural reorganisation. However, as the results of this paper show, ultrasound microscopy appears to be a sensitive tool with which to observe structural alteration of cells following the application of cytotoxic drugs such as INO and $INO_2$. The changes observed with the ultrasound microscope were well-correlated with photomicrographs obtained using conventional optical microscopy, supporting the idea that ultrasound microscopy can be used as a non-invasive approach to study the kinetics of drug action. The length of time required to make the C-scan images shown here (8 min) is another limiting factor. New methods utilising the B-mode of ultrasound imaging in which image data is collected on a line-by-line basis instead of a point-by-point basis, show great promise to eliminate this problem. A scanner based on this principle has been developed by Sherar *et al.* (1989) and tested by Pavlin *et al.* (1991) to image small tumours of the anterior segment of the eye in humans. The time per image in this system is 0.2 s. This type of approach could potentially be used to study drug effects on superficial tumours in animals and humans.

### References

ACKER, H. (1984). Microenvironmental conditions in multicellular spheroids grown under liquid-overlay tissue culture conditions. *Recent Results Cancer Res.*, **95**, 116.

BÉRUBÉ, L.R., FARAH, S., MCCLELLAND, R.A. & RAUTH, A.M. (1991). Effect of 1-methyl-2-nitrosoimidazole on intracellular thiols and calcium levels in Chinese hamster ovary cells. *Biochem Pharmac.*, **42**, 2153.

CARLSSON, J., STÅLNACKE, C-G., ACKER, H., HAJI-KARIM, M., NILSSON, S. & LARSSON, B. (1979). The influence of oxygen on viability and proliferation in cellular spheroids. *Int. J. Radiat. Oncol. Biol. Phys.*, **5**, 2011.

CLARKE, P.R. & HILL, C.R. (1970). Physical and chemical aspects of ultrasonic disruption of cells. *J. Acoust. Soc. Amer.*, **47**, 649.

DOBRUCKI, J.W., DENSAR, F., WALCZAK, T., WOODS, R.K., BACIC, G. & SWARTZ, H.M. (1990). Electron spin resonance microscopy of an *in vitro* tumour model. *Br. J. Cancer*, **61**, 221.

DOBRUCKI, J.W., SUTHERLAND, R.M. & SWARTZ, H.M. (1991). Non-perturbing test for cytotoxicity in isolated cells and spheroids, using electron paramagnetic imaging. *Magn. Res. Med.*, **19**, 42.

FRANKO, A.J., FREEDMAN, H.I. & KOCH, C.J. (1984). Oxygen supply to spheroids in spinner and liquid-overlay culture. *Recent Results Cancer Res.*, **95**, 162.

HALL, E.J. & ROIZIN-TOWLE, L. (1975). Hypoxic sensitizers: Radiobiological studies at the cellular level. *Radiology*, **117**, 453.

LUK, C. & SUTHERLAND, R.M. (1986). Influence of growth phase, nutrition and hypoxia on heterogeneity of cellular buoyant densities in *in vitro* tumor model systems. *Int. J. Cancer*, **37**, 883.

MUELLER-KLIESER, W. (1984). Microelectrode measurements of oxygen tension distributions in multicellular spheroids cultured in spinner flasks. *Recent Results Cancer Res.*, **95**, 134.

NOSS, M.B., PANICUCCI, R., MCCLELLAND, R.A. & RAUTH, A.M. (1988). Preparation, toxicity and mutagenicity of 1-methyl-2-nitrosoimidazole. *Biochem Pharmacol.*, **37**, 2585.

NOSS, M.B., PANICUCCI, R., MCCLELLAND, R.A. & RAUTH, A.M. (1989). 1-methyl-2-nitrosoimidazole: Cytotoxic and glutathione depleting capabilities. *Int. J. Radiat. Oncol. Biol. Phys.*, **16**, 1015.

PAVLIN, C.J., HARASIEWICZ, K., SHERAR, M.D. & FOSTER, F.S. (1991). Clinical use of ultrasound biomicroscopy. *Ophthalmology*, **98**, 287.

RAUTH, A.M. (1986). Pharmacology and toxicology of sensitizers: Mechanism studies. *Int. J. Radiat. Oncol. Biol. Phys.*, **10**, 1293.

SHERAR, M.D. (1990). Ultrasound backscatter microscopy and its application to biological studies. PhD Thesis, Department of Medical Biophysics, University of Toronto.

SHERAR, M.D., NOSS, M.B. & FOSTER, F.S. (1987). Ultrasound backscatter microscopy images the internal structure of living tumour spheroids. *Nature*, **330**, 493.

SHERAR, M.D. & FOSTER, F.S. (1987). A 100 MHz PVDF ultrasound microscope with biological applications. In *Acoustical Imaging*, Kessler, L.W. (ed.) 16, p.511, Plenum: New York.

SHERAR, M.D. & FOSTER, F.S. (1988). Ultrasound backscatter microscopy. *IEEE*, 959.

SHERAR, M.D. & FOSTER, F.S. (1989). The design and fabrication of high frequency polyvinylidene fluoride transducers. *Ultrasonic imaging*, **11**, 75.

SHERAR, M.D., STARKOSKI, B.G. & FOSTER, F.S. (1989). A 100 MHz B-scan ultrasound backscatter microscope. *Ultrasonic Imaging*, **11**, 95.

SILLERUD, L.O., FREYER, J.P., NEEMAN, M. & MATTINGLY, M.A. (1990). Proton NMR microscopy of multicellular tumor spheroid morphology. *Mag. Reson. Med.*, **16**, 380.

SUTHERLAND, R.M. (1988). Cell and environment interactions in tumor microregions: The multicell spheroid model. *Science*, **240**, 217.

TER HAAR, G.R. & STRATFORD, I.J. (1982). Evidence for a non-thermal effect of ultrasound. *Br. J. Cancer*, **45**, 172 (suppl. V).

WYLLIE, A.H. (1987). Apoptosis: Cell death in tissue regulation. *J. Pathol.*, **153**, 313.